# Vascular access for initial treatment of adult emergency patients in the resuscitation room: short summary of recommendations from the German national S1 guideline

**DOI:** 10.1186/s12245-025-01015-x

**Published:** 2025-10-02

**Authors:** Manuel Florian Struck, Dan Bieler, Anett Henck, Carsten Hermes, Michael Kegel, Matthias Klein, Philipp Kümpers, Dominik Michalski, Michael Bernhard

**Affiliations:** 1 Klinik und Poliklinik für Anästhesiologie und Intensivtherapie, Universitätsklinikum Leipzig, Liebigstr. 20, 04103 Leipzig, Germany; 2 Klinik für Unfallchirurgie und Orthopädie, Wiederherstellungs- und Handchirurgie, Verbrennungsmedizin, Bundeswehrzentralkrankenhaus Koblenz, Koblenz, Germany; 3 Klinik für Anästhesiologie, Universitätsklinikum Heidelberg, Heidelberg, Germany; 4 Städtisches Klinikum Karlsruhe gGmbH, Karlsruhe, Germany; 5 Hochschule für Angewandte Wissenschaften, Hamburg, Germany; 6 Akkon Hochschule für Humanwissenschaften, Berlin, Germany; 7 Bildungsakademie der Gesundheit Nord gGmbH, Klinikverbund Bremen, Bremen, Germany; 8 Neurologische Klinik und Poliklinik, Klinikum der Ludwigs-Maximilian- Universität München, München, Germany; 9 Sektion Interdisziplinäre Notaufnahme, Medizinische Klinik D, Zentrum für Klinische Akut und Notfallmedizin, Universitätsklinikum Münster, Münster, Germany; 10 Klinik und Poliklinik für Neurologie, Universitätsklinikum Leipzig, Leipzig, Germany; 11 Zentrale Notaufnahme, Universitätsklinikum Düsseldorf, Düsseldorf, Germany

**Keywords:** Vascular access, Peripheral intravenous line, Central venous catheter, Arterial line, Resuscitation room, Emergency department

## Abstract

The initial treatment in the resuscitation room involves vital procedures, including the placement of peripheral intravenous lines, central venous catheters, and arterial lines. As most vascular access guidelines were not developed for resuscitation room settings, clear recommendations were necessary. This guideline has been developed by an interdisciplinary, interprofessional group of experts from five German national medical professional societies. The first part of the guideline provides general recommendations for vascular access in the resuscitation room in adults, whereas the second part describes specific recommendations and strategies for particular emergency situations.

## Background

The initial treatment in the resuscitation room involves vital procedures, including the placement of peripheral intravenous lines (IV), central venous catheters (CVCs), and arterial lines. A variety of strategies can be employed, ranging from swiftly establishing these vascular accesses to stabilize the patient, to avoiding CVC and arterial line placements in favor of rapid, computed tomography (CT) based diagnostics to detect the underlying causes of the present symptoms, which may have direct consequences. For instance, this could involve suspected acute pulmonary embolism and the initiation of thrombolytic medication, or confirming intracranial hemorrhage and scheduling subsequent decompression neurosurgery. The placement of central venous access or arterial blood pressure measurement in the resuscitation room are invasive interventions requiring the correct indication and experienced operators while responding to life-threatening conditions quickly and appropriately. While most vascular access guidelines are not developed for the framework conditions of emergency departments, it was necessary to formulate clear recommendations for resuscitation room settings.

## Methods

This guideline is based on the consensus of an interdisciplinary and interprofessional group of experts led by the German Society of Anesthesiology and Intensive Care Medicine (DGAI) and including members mandated by the German Society of Interdisciplinary Emergency and Acute Medicine (DGINA), the German Society for Internal Intensive Care and Emergency Medicine (DGIIN), the German Society of Neurointensive and Emergency Medicine (DGNI), and the German Society of Trauma Surgery (DGU). The guideline was developed in accordance with the formal requirements of the German Association of the Scientific Medical Societies (AWMF) for an S1 recommendation level. In Germany, guideline recommendations are published by the AWMF and classified according to different levels of evidence. Level S1 is primarily expert recommendation in the absence of high-quality studies (randomized controlled trials, RCTs). Levels S2k and S2e are consensus-based (representative board and structured consensus process) and evidence-based (systematic search, selection, and classification of existing literature) guidelines, respectively. The highest level S3 is both consensus-based and evidence-based (including the availability of a large body of RCTs).

The results of a selective literature review were analyzed, discussed and evaluated in a total of eight consensus meetings, from which the core statements and recommendations now available were formulated [1].

However, the number of randomized controlled trials available for establishing vascular access and invasive arterial blood pressure measurements in the resuscitation room remains limited. Accordingly, the literature supporting this guideline is considered to have only weak evidence categories, as defined by the Agency for Health Care Policy and Research (AHCPR) and the Scottish Intercollegiate Guidelines Network (SIGN). The dearth of studies in this area is primarily attributable to the unique characteristics of the emergency medical setting, the wide range of patient illnesses and injuries, the local and structural conditions of emergency departments, and the often differing experiences and standards in the use of vascular access in emergency situations. The first part (A) of the guideline offers general recommendations for vascular access in the resuscitation room. The second part (B) outlines specific recommendations and concepts for designated emergency scenarios. Some key references of the previously published original edition (in German) have been provided for better understanding.

## Results

A) General recommendations
  1. Indication for vascular accesses
    At least two large-lumen peripheral venous access sites with a flow rate of at least 100 ml/min (e.g. 18G) should be available in the resuscitation room.
    After examination, vascular accesses placed by prehospital emergency medical services (EMS) can be used as well.
    In the event of an immediate life-threatening condition and failed peripheral venous access, an intraosseous access should be established. This is particularly important in the case of cardiac arrest, in which IO access should be established immediately when quick IV access is not possible [2].
    Continuous administration of vasopressors can be safely performed via peripheral venous access if the catheter is safely intravascular, the infusion arm is calmly positioned, and a low-concentration solution is used [3, 4].
    In cases where there is a pronounced need for vasopressors after stabilization via peripheral venous access (e.g., due to ongoing bleeding or anesthetics-related hypotension), a CVC can be placed following initial diagnostic imaging (e.g. CT) before transfer to the intensive care unit (ICU) or the operating room.
    A CVC should be placed if there is insufficient peripheral venous access and if an unstable circulatory situation requiring immediate stabilization is foreseeable. The specific type of central venous access required (e.g., three-lumen CVC, hemodialysis catheter, or introducer sheath) should be considered depending on the underlying disease or injury.
    Critically ill or severely injured patients should undergo arterial blood pressure measurement.
    In cases of compensated circulatory conditions, placement of a CVC and an arterial line should not delay initial diagnosis (e.g. CT).
  2. Hygienic measures
    The standard hygiene guidelines should be observed, taking into account the type of catheter used [5, 6].
    In cases where aseptic vascular access is not possible due to emergency conditions, catheters should be removed and placed at a different location within 24 h. Prophylactic intravenous administration of antibiotic medication for vascular access is not indicated.
  3. Sonographic guidance and puncture
    Sonography can improve the success rate of puncturing peripheral veins in patients with difficult peripheral veins, and when inserting CVCs and arterial lines [7].
    CVC placement should particularly be performed under sonographic guidance whenever possible [8–12].
    Operators should nevertheless be able to perform CVC placement via the landmark-guided technique when sonography devices are unavailable.
  4. Position confirmation and functionality check
    Peripheral venous access and all CVC lumens should be aspirated and flushed with sterile 0.9% sodium chloride solution before use. They should also be checked for leakage or extravasation.
    Arterial catheters should be aspiratable and flushable and able to generate a typical arterial blood pressure curve.
    Arterial CVC malpositions can be detected by connecting to a pressure system and obtaining an arterial blood pressure curve.
    Catheter tips of thoracocervical CVCs can be verified using endovascular electrocardiography (ECG) if a sinus rhythm is present [10].
    CVC position can be verified at the bedside using transthoracic echocardiography and the administration of a fluid bolus via the CVC (‘rapid atrial swirl sign’) [13].
    Acute diagnostics (e.g. initial polytrauma CT) can be used to verify the correct position of the CVC [14].
  5. Fixation and securement
    All vascular access points should always be securely fixed.
    Special fixation sets provided by the manufacturers should be used instead of improvised solutions, especially for large-lumen cannulas (e.g., extracorporeal membrane oxygenation, ECMO).
    All CVCs and femoral arterial access points should be secured with a suture.
    Catheters in distal arteries, usually in the radial artery, should not be sutured as standard.
    CVC securing sutures should be applied to the catheter´s distribution hub and not just to the clamps [15].
    CVC insertion depth should be documented on the dressing and in the protocol.
  6. Mechanical complications
    Vascular access procedures, as well as any puncture-related difficulties or mechanical complications, should be documented in writing [16].
B) Special recommendations
  1. Patients with severe injuries and multiple trauma/polytrauma
    In cases of cervical spine immobilization, CVC placement via the internal jugular vein is impractical.
    In cases of severe thoracic injury or the presence of a chest tube, it is advisable to place a CVC via the subclavian vein on the injured/drained side.
    Depending on the presence and position of a pelvic binder, femoral double puncture of the ipsilateral artery and vein may be a viable option [12].
    In cases involving a resuscitative endovascular balloon occlusion of the aorta (REBOA) system, the standards for puncture and sheath size should be considered.
    In patients in extremis, for whom an inguinal pulse cannot be palpated, vascular access should be performed with ultrasound guidance or surgically (cut-down).
    In severely burned patients, CVC and arterial line placement for cardiac output measurement/transpulmonary thermodilution can be performed in the resuscitation room or during the admission bath, depending on circulatory stability and the extent of the burns, to guide fluid resuscitation.
  2. Patients with cardiovascular emergencies and respiratory insufficiency
    In cases of respiratory insufficiency, puncturing the subclavian vein should be avoided in order to prevent iatrogenic pneumothorax. Internal jugular or femoral access may be preferable when feasible. If subclavian access is unavoidable, ultrasound guidance and pleural scanning can reduce the risk (i.e. the axillary approach).
  3. Patients with neurotrauma, unconsciousness und cerebrovascular incidents
    Upper body positioning at an elevation of 15–30° is recommended for patients with increased intracranial pressure. This position should be maintained if a CVC is required [17, 18].
    Currently, there is no clear evidence supporting the idea that a jugular CVC would cause significant impairment of venous outflow. A subclavian CVC should be avoided if thrombolytic or anticoagulant substances need to be administered to prevent puncture-related thoracic bleeding complications. Therefore, we recommend the left femoral approach instead (reserving the right femoral approach for angiographic procedures).
  4. Sepsis and multiorgan dysfunction
    Fluid therapy should be guided by arterial and central venous monitoring.
    In patients with overt sepsis, hyperkalemia, and/or acute renal failure, a dialysis catheter can be inserted in the resuscitation room to rapidly initiate renal replacement therapy.
  5. Cardiac arrest
    Patients who have experienced a return of spontaneous circulation (ROSC) or who are undergoing cardiopulmonary resuscitation (CPR) should first receive an arterial blood pressure measurement following peripheral venous access. However, establishing arterial access for invasive blood pressure measurement should not cause a delay to life-saving or diagnostic procedures.
    In cases involving extracorporeal CPR (ECPR), the in-house standards for vessel cannulation should be considered.
    CPR patients with intraosseous access only should also receive a peripheral venous cannula [2, 19].

## Conclusion

Providing emergency vascular access in the resuscitation room is often challenging and requires clinical expertise and knowledge of the different requirements of various emergency situations. This is the first national guideline on emergency vascular access specifically designed for resuscitation rooms. While it is primarily based on expert consensus rather than randomized controlled trials, the objective is to provide clinicians with practical guidance for daily resuscitation room care. Further research in this area is necessary to enhance the treatment and safety of emergency patients.

## Data Availability

The initial guideline was published in German language by the AWMF [1] under the coordinating national medical professional society of DGAI, which plans to publish another German version of the guideline.

## Abbreviations

AHCPR: the Agency for Health Care Policy and Research
AWMF: the German Association of the Scientific Medical Societies
CT: Computed tomography
CVC: Central venous catheter
CPR: Cardiopulmonary resuscitation
DFG: the German Research Association
DGAI: the German Society of Anesthesiology and Intensive Care Medicine
DGINA: the German Society of Emergency Medicine (formerly the German Society of Interdisciplinary Emergency and Acute Medicine)
DGIIN: the German Society for Internal Intensive Care and Emergency Medicine
DGNI: the German Society of Neurointensive and Emergency Medicine
DGU: the German Society of Trauma Surgery
ECG: Electrocardiography
ECMO: Extracorporeal membrane oxygenation
ECPR: Extracorporeal cardiopulmonary resuscitation
EMS: Emergency medical service
IO: Intraosseous
IV: Intravenous
REBOA: Resuscitative endovascular balloon occlusion of the aorta
ROSC: Return of spontaneous circulation
SIGN: the Scottish Intercollegiate Guidelines Network
